# ‘When hunger makes everything better looking!’: The effect of hunger on the aesthetic appreciation of human bodies, faces and objects

**DOI:** 10.1186/s40359-022-00807-7

**Published:** 2022-04-11

**Authors:** Valentina Cazzato, Carmelo M. Vicario, Cosimo Urgesi

**Affiliations:** 1 School of Psychology, Faculty of Health, Liverpool John Moores University, Liverpool, UK; 2 Department of Cognitive, Psychological, Pedagogic Sciences and of Cultural Studies, University of Messina, Messina, Italy; 3 Department of Languages and Literatures, Communication, Education and Society, University of Udine, Udine, Italy; 4 Scientific Institute, IRCCS E. Medea, Bosisio Parini, Lecco Italy

**Keywords:** Fasting, Snack, Body mass index, Body appreciation, Appetite

## Abstract

**Background:**

Research evidence suggests that physiological state of hunger might affect preference for female body weight, such that hungrier, compared to satiate, men prefer heavier body weight and rate as more attractive heavier female figures. Here, we seek to extend these findings by comparing the effects of fasting and snack on aesthetics judgements of the bodies and faces of conspecifics and of objects in a sample of female and male participants.

**Methods:**

Forty-four participants (women: n = 21, mean age = 23.70 yrs ± 0.62) provided aesthetic liking judgments of round and slim human bodies, faces and objects, under at least 12 h of overnight fasting and immediately after having eaten a snack (i.e., bananas). An anthropometric measure of adiposity (i.e., Body Mass Index, BMI) was also collected from each observer.

**Results:**

Overall, we found that participants’ aesthetic judgements were higher for slim stimuli compared to round ones. However, after fasting, participants rated round stimuli as more attractive compared to when they had a snack. This hunger-based shift in ratings not only was apparent when stimuli depicted a human body or face, but also when they depicted an object, thus suggesting a general modification of observers’ aesthetic preference related to hunger. Importantly, this effect was modulated by participants’ BMI so that only participants with a high BMI provided higher aesthetic judgements for round stimuli after fasting than after a snack.

**Conclusions:**

Our results demonstrated that both the modification of the physiological state and the individual differences in adiposity level of the observers might affect the aesthetic appreciation of the external world.

## Introduction

Hunger offers the strongest homeostatic motivation for behaviour in the animal kingdom, including humans. From an ethological perspective, animals must select and pursue food in times of caloric insufficiency, despite the wide diversity of stimuli and competing demands that naturally have a bearing on them. To address this homeostatic imbalance, animals must be able to successfully navigate their environment in ways that require them to flexibly switch between exploratory, defensive, and competing behaviours, indicating tremendous plasticity in feeding behaviour. Interestingly, a relevant study on mice supports this notion by reporting that, when mice are motivated to pursue other needs, such as water consumption, self-preservation in fear-inducing contexts, or social interaction with conspecifics, hunger overrides competing incentives to promote feeding behaviour [1].

Despite humans might more likely, as compared to other animals, to abstain from eating for a short period of time, for example for religious compliance or weight-loss dieting, even when access to food might not be that limited, hunger is nevertheless a primary need that takes priority in perception, action and cognition. It is known that calorie intake reduction and associated sensations of hunger have an impact on human cognition, including attention, memory, and executive function (see [2] for an extensive review). In particular, it has been widely reported that fasted individuals are more attracted to food [3] and more motivated to food acquisition and consumption [4] as compared to satiated ones. However, they are also more attracted to money [5], which can let them to acquire food, and have greater desire to possess various objects, to acquire non-food items, and also to take more samples of non-food objects [6]. Thus, hunger seems to lead to a general increase of acquisition-related attitudes and behaviours, influencing domains that are not relevant for reducing hunger [6]. In a similar vein, an observer’s level of hunger may also influence the perception of others’ actions when they are directed to not only a primary- (e.g., a muffin) or secondary-reinforced (i.e., a banknote) object, but also to a neutral object (e.g., a notepad; [7]). Finally, there is evidence that hunger can affect human decision-making in the field of morality, by reducing disapproval of ethical violations (e.g., [8, 9]).

Interestingly, researchers have also reported the impact of hunger on other types of decisional processes such as the appreciation of human physical attractiveness, with most of them focussing on women’s bodies and on men’s ratings. The focus of previous studies on men’ aesthetic judgements of women’ bodies might be related to the importance of female body weight as an indicator of fertility, desirability, and sexuality [10], as well as a sign of health, endurance, wealth, and a reflection of a higher social position [11]. Indeed, many reviews [12–15] on food insecurity and body weight all report a positive association between food insecurity and high body weight in women, while the association is less clear or absent in men [16]. In a similar vein, a series of studies of female attractiveness demonstrated higher level of consistency in men’ preferences for female bodies across individuals, cultures, and experimental techniques [17–19]. On the contrary, women’ judgements of male attractiveness are consistent with a trade-off between cues to fertility and to expected paternal investment. With these regards, it has been shown that female preferences may change in response to personal circumstances, but wider scale environmental, cultural, and ethnic factors may also influence the balance of the trade-off [20], thus making their judgements less consistent.

Evidence to suggest a link between appetite and body image perception has been reported by a series of studies by Swami and Tovée [21] and Nelson and Morrison [22]. In their study, Nelson and Morrison [22] showed that men who were about to eat an evening meal rated as more attractive a heavier female body weight compared to men who had just eaten an evening meal, whereas women’s rating of men’s ideal body weight did not vary with those women’s meal status. Swami and Tovée [21] asked hungry and satiated participants to rate a series of photographs of women with known body weight and shape. Corroborating the previous findings from Nelson and Morrison’s study [22], their results indicated that before dining men preferred slightly heavier women (measured in terms of their Body Mass Index, BMI) than after dining. Notably, hungrier men also rated overweight and obese women more favourably. In keeping with these findings, a further study by Pettijohn, Sacco and Yerkes [23] asked men to select the weight category in pounds of an ideal female partner and found that hungry men gave more positive ratings to heavier, as compared to slimmer women.

On the other hand, a recent randomized, controlled study by Jin and colleagues [24] failed to replicate previous non-randomized observational studies, which supported ratings of female physical attractiveness provided by males are sensitive to levels of hunger. Two studies were conducted. In the first study, a sample of male participants were recruited and after an overnight fast they were randomly allocated to either fed or starved conditions: one group was left to starve, while a second group was given ad libitum access to foods and was encouraged to eat to full satiation. Four hours later, participants were asked to complete a female attractiveness rating test, along with other cognitive tasks. Hunger levels were monitored using both subjective measures of hunger and objective measures of levels of circulating glucose. No effects of hunger were found on the judgements of body attractiveness. In the second study, which was a double-blind experiment, a subgroup of participants was recruited to evaluate if the original effect was due to a confounding impact of alcohol consumption when dining. Circulating alcohol levels were quantified by a breath test, and they repeated the female attractiveness rating test. Results showed a significant negative relationship between circulating alcohol and the mean adiposity of individuals rated as least attractive, thus suggesting that the reason for the difference from previous studies might be ascribed to the fact that in previous studies hunger was possibly confounded by alcohol consumption.

All of these converging results (except for [24]) have been interpreted in light of the so called ‘Insurance Hypothesis’ [25]. According to this view, individuals who experience scarcity of resource, as when they feel hungry, respond to body adiposity as a protection from food scarcity. This shifts the aesthetic preference toward rounder body figures [21, 22, 26, 27]. The relation between the experience of hunger and a more favourable perception of body adiposity, however, would predict that only the aesthetic judgment of human bodies is affected by hunger, while the perception of other objects should not. In keeping with this prediction, a series of studies by Swami et al. [26] asked hungry men to provide aesthetic judgments for a series of images of anvils or empty milk bottles of different sizes, or of milk bottles of different fill levels (empty to full) and did not find evidence to suggest that hunger influenced judgements of these other types of objects. Accordingly, Xu et al. [6] found that while hunger increased both the intention to acquire and the liking of food items, it promoted only the acquisition, but not the liking of non-food objects. However, Saxton et al. [27] have recently tested the effects of self-reported feeling of hunger on the attractiveness judgements of female and male bodies manipulated to vary in level of adiposity and of objects manipulated to vary in size. They found that, even if the effects of hunger were greater for bodies, larger sizes of stimuli in general were judged as more attractive by hungrier participants, thus questioning the body-selective effects of hunger on aesthetic preference.

Nevertheless, correlating individual sense of hunger in uncontrolled situations rather than manipulating food intake condition might confound hunger signals with interindividual variabilities in the experience and report of hunger. Indeed, people may differ in their experience of hunger for many reasons. For example, individual differences in the interoceptive states of hunger might concern the ability of detecting visceral signals and their changes, as well as the experience of differing motivational, affective and cognitive states under similar physiological input (see [28] for an extensive review on this topic). Personality traits such as cognitive restraint, which is the degree to which a person consciously regulates their food intake, may also mediate attention to interoceptive state of hunger [29, 30]. With these regards, a previous study by Lattimore [31] suggested that adiposity, as measured by BMI, rather than dietary restraint might be an important mediator of appetite-specific body dissatisfaction and aesthetic preference. Accordingly, the study by Jin and colleagues [24] showed that it was not the starved or fed condition to which two groups of participants were randomly allocated, but subjects’ BMI that influenced ratings of female attractiveness. In fact, a strong negative relationship between ratings of attractiveness and BMI of the subject was reported and this relationship did not differ between fasted and fed groups.

To further address the specificity of the effects of hunger on body weight perception, here, we devised a within-subject, lab-based study gathering aesthetic ratings of body and object stimuli in both male and female participants. Crucially, we also included another type of body-related stimuli, namely faces, which can serve as a proxy for the estimation of facial adiposity [32]. Finally, we included a neutral (non-corporeal, non-food stimuli) object, a vase. The use of vases as control object in body perception is not new. Indeed, a study by McCabe et al. [33] used a vase to control for perceptual estimation errors due to factors unrelated to the perception of one’s own body. This way, the inclusion of the non-food stimuli vase acted as a control for perceptual errors inherent in the task, but unrelated to body size [33]. Accordingly, to avoid any bias related to the presentation of food-related stimuli, whilst also presenting aesthetically pleasing stimuli, we decided to include non-food stimuli vases so to establish whether hunger-based shift ratings of round and thin figures are specific for bodily stimuli or might also extend to faces or other non-bodily objects. Particularly, we aimed to investigate whether physiological conditions of fasting and snack might affect participants’ aesthetic ratings of liking when judging the appearance of thin and round bodies and faces, as compared to a familiar, control object (i.e., a vase).

To address the limitations of between-subject [21–24, 26] or correlational [27] designs employed by the previous studies and to control for interindividual differences in the experience of hunger, the same participants provided aesthetic judgements of stimuli under two different physiological conditions (in two different days, counterbalanced between participants): while being in food deprivation (i.e., after at least 12 h of fasting) and after having consumed a snack (i.e., after at least 12 h of fasting). Importantly, in each physiological condition, participants were asked to provide their subjective visual analogue scale (VAS) hunger ratings with the aim to disentangle the effect of food deprivation/intake from the effect due to changes in the hunger visceral sensation associated with the two physiological conditions [7, 8]. Finally, we also measured participants’ BMI that is known to affect hunger perception and physical attractiveness [7, 24, 34].

Capitalising on previous literature, we expected our participants to provide higher liking judgments to slim than to round models [35–37]. However, we anticipated this effect to be influenced by participants’ physiological state of hunger, so that participants will provide higher liking ratings to round stimuli when they are hungry as compared to when they are satiated. More specifically, in line with the predictions of the Insurance Hypothesis’ [25] and previous related findings [21, 22, 26], we expected that the effects of hunger should be specific for those stimuli that convey information about body adiposity, mainly bodies and, at least partially, faces, but not for object size, which is unrelated to body adiposity. Conversely, if hunger exerts a domain-general bias in bountifulness appreciation, shifting preferences toward bigger stimuli in general [27], then comparable effects of hunger should be obtained for all object categories. Finally, we also expected perceived hunger and individual BMI to moderate the effects of the physiological status of hunger with greater increase of aesthetic appreciation of round stimuli in hungrier [22, 27] and heavier [7, 31] participants.

## Methods

### Participants

The sample size for our 2 × 3 × 2 ANCOVA design (numerator *df* = 2; 3 covariates) was based on a preliminary calculation using the freely available G*Power software (G*Power 3.1.9; [38]), which indicated a minimum sample of 44 participants as adequate for a design with 85% power to detect a large effect size (*f* = 0.52), with alpha at 0.05 (two tailed). The effect size was estimated by averaging the effect size of the two studies reported in Nelson and Morrison ([22] study 1, *ηp*^2^ = 0.017; study 2, *ηp*^2^ = 0.018) and that reported by Swami and Tovee’ ([21]; *ηp*^2^ = 0.6). A total of 44 participants (women: n = 21, mean age = 23.70 yrs ± 0.62; mean BMI = 23.25 kg/cm^2^ ± 0.49) from Liverpool John Moores University (LJMU) participated in the experiment in return for course credits or £5 shopping vouchers. All subjects but eight were right-handed as assessed by the Edinburgh Handedness Inventory [39]. Participants were asked to provide their self-identified gender identity (and not their biological sex), which was assessed through two forced-choice boxes (female, male). Participants (self)reported normal or corrected to normal vision and they were in good health, were free of psychotropic or vasoactive medication, with no current or history of psychiatric or neurological disease. Participants reporting specific diet requirements and/or allergies to plantain-based foods, being pregnant or diabetic (self)excluded from this investigation. All participants gave their written informed consent, and all were debriefed at the end of the experiment. All procedures were approved by the university research ethics committee of LJMU, in agreement with the ethical standards of the 1964 Declaration of Helsinki.

### Experimental stimuli

**Fig. 1:**
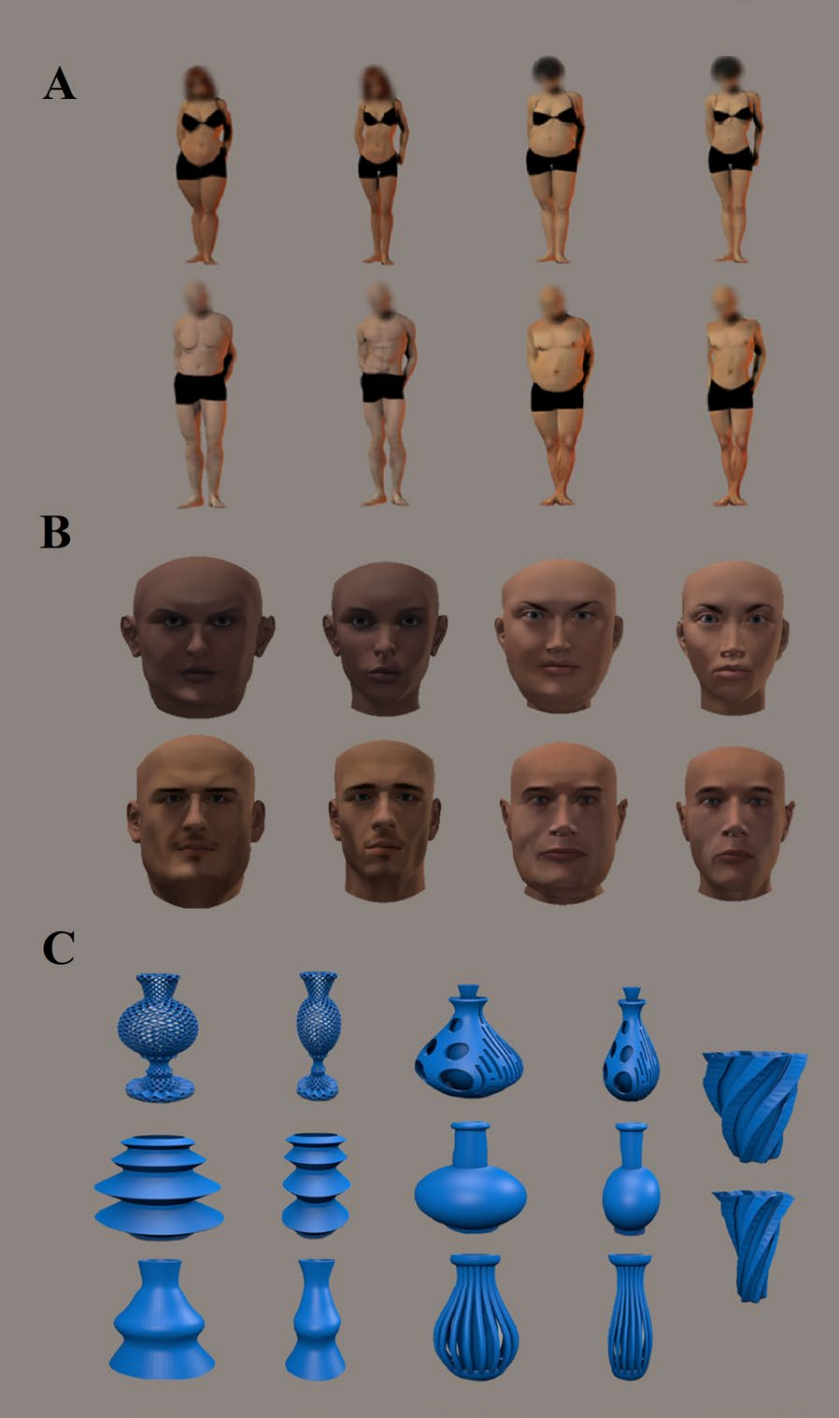
Examples of body (**A**), face (**B**), and object (**C**) stimuli used during the aesthetic (liking) task. The slimmest and largest stimulus of each type is displayed.

Computer-generated imagery (CGI) was used to create 3D stimuli of bodies, faces and familiar objects (i.e., vases), which were designed by means of Poser Pro 2010 (e-frontier, Santa Cruz, CA) and were taken from the stimulus set of a previous study by our group (see [35] for specific details). 3D body stimuli consisted of two female and two male models, and they were wearing identical underwear black clothing. All models were standing against a grey background. Furthermore, each body model was displayed in different postures, from a frontal or three-quarter perspective. The apparent body weight of each model varied according to two levels of round and thin figures. In all body stimuli, the face was scrambled to rule out the impact of face identity discrimination during aesthetics judgements (see Fig. 1A).

Like the body stimuli, 3D facial stimuli consisted of two female and two male models, taken from a previous study by our group [34] and selected to match liking ratings of body stimuli. Faces were depicted against a grey background. We also removed the hair to avoid any confound on perceived attractiveness. Each 3D model face displayed two different neutral expressions and was viewed from a frontal or three-quarter perspective. The level of adiposity of the faces was also manipulated to depict two levels of round and thin appearance (see Fig. 1B).

Finally, for the object stimuli, we used virtual objects that depicted seven different virtual exemplars of vases. To match our object stimuli with the body and face stimuli perceptual features, each of these vases was presented from two different views and rendered against a grey background. Crucially, the size of each object exemplar was altered to reflect two levels of round and thin shape (see Fig. 1C).

Body, face and object stimuli were presented in three separate blocks. The order of the blocks was counterbalanced between-participants, but it remained the same for the two hunger conditions of the same participant. Before the start of each experimental block, participants were introduced to the aesthetic task and presented with five practice trials (taken from a different set of body, face and object stimuli to avoid familiarisation), which were not considered in the main analyses. Each block was composed by two sub-blocks, each comprising 32 slim and 32 round stimuli, which were randomly presented (for a total of 128 stimuli per block). The participants could rest between blocks for how long they needed, usually no longer than 60 s.

### General procedure

The experiment was performed by means of E-Prime software (version 2.0 Professional, Psychology Software Tools, Inc., Pittsburgh, PA) running on a Windows PC. During the experimental sessions, all participants were required to seat approximately 57 cm in front of a 15.6‐inch LCD monitor (resolution, 1024 × 768 pixels; refresh frequency, 60 Hz), and were asked to complete an aesthetic (Liking) task. During each trial, participants were presented with a black central fixation cross on a grey background. After 500 ms, a body (or face or object) appeared for 500 ms at the center of the screen subtending a visual angle of approximately 12° × 10°. Then, the stimulus was replaced by a random-dot mask obtained by scrambling the corresponding sample stimulus by means of custom-made image segmentation software (Matlab 9.5, The Mathworks, Inc., Natick, MA, USA) which lasted on the screen for 500 ms. Finally, participants were prompted to provide their ratings by answering to the question ‘How much do you like this body (or face or object)?’ which also appeared at the center of the screen. The question was always presented along with a horizontal, 10 cm VAS ranging from ‘very much’ (100) to ‘not at all’ (0). The right- and left-side position of the anchor words of the VAS was balanced across participants. In keeping with previous studies of body aesthetic experiences [35–37, 40, 41], addressing the ‘Liking’ dimension allowed us to focus on the subjective, individual attribute concerning the observer toward the stimulus or its features as compared to a more objective, shared regarding the perceptual properties of the stimulus (beauty).

Based on the successful experimental manipulation of hunger adopted by the study of Vicario and colleagues [7], we required participants to complete the aesthetic task in two different occasions, with each one corresponding to two different days. The two visits were always scheduled at morning time (i.e., between 08:00 and 11:30 am) after at least a break of 24 h in between. During the two visits, participants were required to eat their last meal at least 12 h before the scheduled sessions. During a fasting session, participants completed the aesthetic task after at least 12 h of overnight fasting; during a snack session, participants completed the aesthetic task immediately after having eaten a snack (i.e., bananas). Participants were allocated to one or the other order of physiological status condition in a random manner. The order of fasting/snack sessions among participants was counterbalanced, so that half of participants started the first session after at least 12 h of overnight fasting but before having eaten the snack (fasting session); and the second session after at least 12 h of overnight fasting but immediately after having eaten the snack (snack session). The other half of participants completed the task in the reversed order. Participants were invited to eat the snack until they felt satiated (for a maximum of two bananas). Following this, they were asked to provide hunger ratings both before and after breaking the fast using a horizontal 10-cm VAS, with anchor points labelled ‘‘Not at all hungry’’ to ‘‘Extremely hungry’’. During the first visit, before starting the experimental tasks, anthropometric measure of adiposity, that is Body Mass Index (BMI; kg/cm^2^), was obtained from measuring weight (kg) and height (cm), by means of a scale and a stadiometer. Overall, testing lasted approximatively 45 min per session.

### Data handling

All statistical analyses were performed using STATISTICA 8.0 (StatSoftInc, Tulsa, Oklahoma). First, a series of two-tailed t-tests were performed to compare appetite ratings in the fasting and the snack conditions. Second, the mean Liking VAS scores were entered in a 2 (fasting, snack-physiological status) × 3 (body, face, object-stimuli) × 2 (round, slim-size) repeated measures analyses of covariance (ANCOVA), entering participants’ BMI, appetite ratings in the fasting and the snack conditions as continuous predictors. This way, we could test not only the effects of food depletion and intake (i.e., in association to fasting vs. snack condition), but also those of the individual perceived level of hunger in each condition. Whenever a covariate showed significant interactions with the other variables, we used a median-split procedure to assess the effects of these within-subjects variables (and their interaction) on the mean Liking VAS scores at particular levels of the covariate using a repeated-measure ANOVA design [42], separately for individuals with covariate scores below vs. above the median. Finally, a bivariate correlation between participants’ BMI and change in appetite [(RoundFasting – RoundSnack) – (SlimFasting – SlimSnack)] for all three stimuli was calculated to ascertain the relationship between BMI and the interaction effect. All data are reported as Mean (M) and Standard Error of the Mean (S.E.M.). A significance threshold of *p* < 0.05 was set for all effects and effect sizes were estimated using the partial eta square measure (*ηp*^2^). Duncan post-hoc tests were performed to follow-up significant interactions.

## Results

Manipulation of hunger and satiety worked as expected. Indeed, the two tailed t tests showed significant higher self-reported hunger ratings in the fasting condition (60.15 ± 25.03), compared to the snack (39.94 ± 29.48) condition [*t*_(43)_ = 4.35, *p* < 0.001].

### Aesthetic task

The ANCOVA on the liking ratings revealed a main effect of size, which indicated that, overall, slim stimuli were liked more that round stimuli [*F*_(1,40)_ = 11.014, *p* = 0.002, *ηp*^2^ = 0.216]. The main effect of type of stimuli was moderated by the covariate of self-reported hunger ratings for the snack condition [*F*_(2,80)_ = 4.270, *p* = 0.017, *ηp*^2^ = 0.096]. This suggests that the experimental manipulation of the physiological status (snack condition) had different effects on the aesthetic judgements of the three types of stimuli according to the subjective levels of perceived hunger of participants. To explore the source of the significant modulation of participants’ hunger ratings on the main effect of type of stimuli, we split participants into those with a hunger rating below the median (Low; n = 19]) and those with a hunger rating above the median (High; n = 25]), based on participants’ median hunger ratings (median hunger ratings = 30). Therefore, we conducted two separate follow-up one-way ANOVAs with type of stimuli as within subject variable, respectively for Low vs. High hunger ratings’ groups. The ANOVAs revealed a significant main effect of type of stimuli for both Low hunger [*F*_(2,36)_ = 6.013, *p* = 0.006, *ηp*^2^ = 0.250] and High hunger ratings groups [*F*_(2,36)_ = 5.826, *p* = 0.005, *ηp*^2^ = 0.195]. Post hoc analysis showed that amongst Low hunger participants, body stimuli were the most liked, compared to faces and objects stimuli (50.42 ± 1.63, all *p*_*s*_ < 0.025). No difference was observed between the liking ratings of faces and objects (42.53 ± 2.17 vs. 44.95 ± 2.48, *p* = 0.305). For High hunger participants, body and objects were rated as equally likable (46.26 ± 1.40 vs. 45.67 ± 1.46, *p* = 0.658). On the other hand, faces were liked the least (42.09 ± 1.73, all ps < 0.004).

**Fig. 2:**
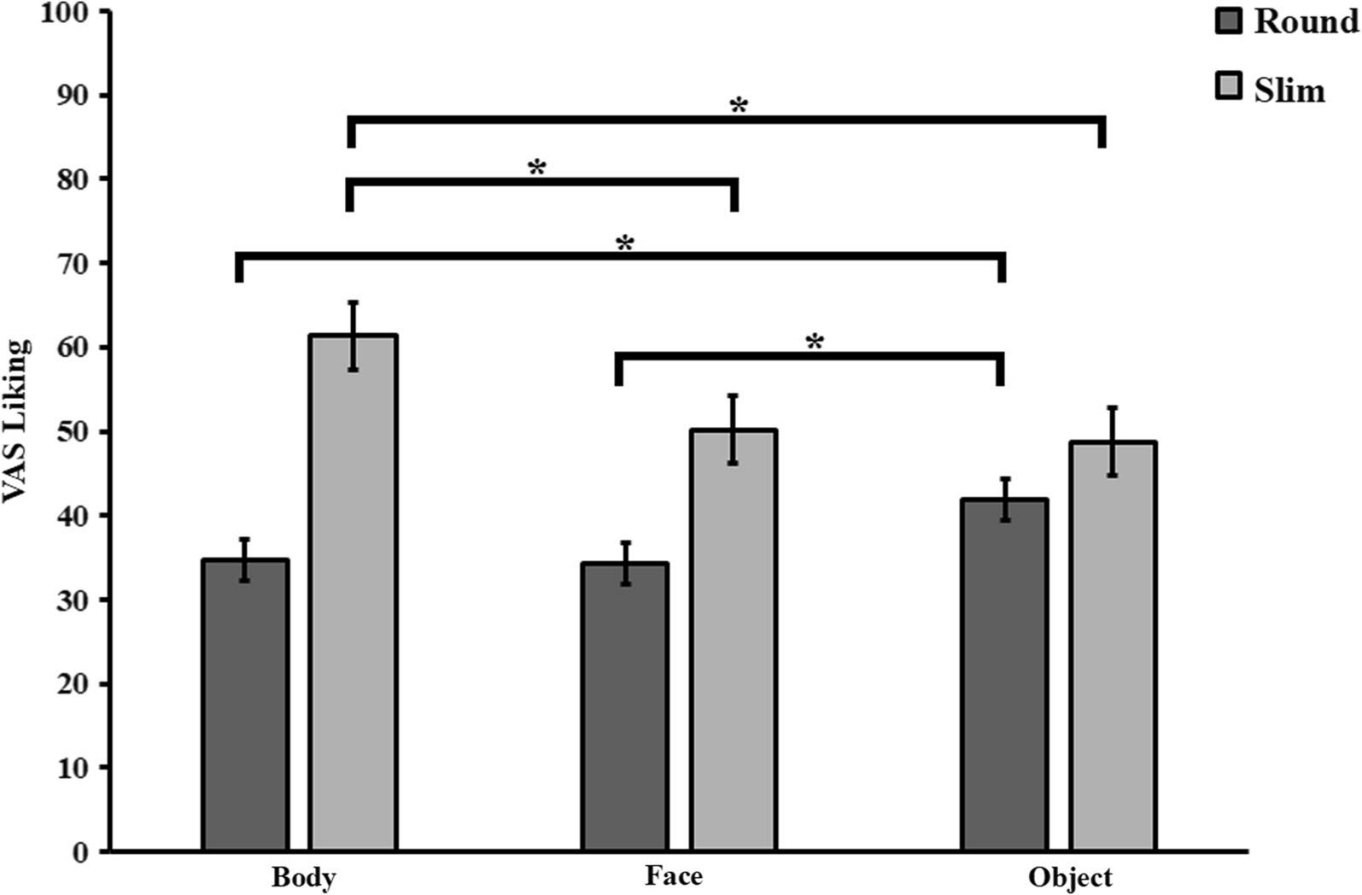
Mean (±SEM) scores of the visual analogue scale (VAS) of the aesthetic judgements for the three types of stimuli (bodies, faces and object) for the two levels of roundness (round, slim). The asterisk symbol indicates a significant difference (*p < 0.05).

A two-way interaction of type of stimuli × size was observed [*F*_(2,80)_ = 5.178, *p* = 0.008, *ηp*^2^ = 0.115, see Fig. 2]. Post hoc analyses revealed that round stimuli were always liked less than slim stimuli (all *ps* < 0.001). However, round objects were liked significantly more compared to round bodies and faces (all *ps* < 0.001), whose appreciation in turn did not differ (*p* = 0.771). Conversely, slim bodies were liked significantly more than slim faces and objects (all *ps* < 0.001), whilst no significant difference in the liking ratings was observed between slim faces and objects (*p* = 0.287).

Notably, the significant main effect of size was also moderated by the covariate of participants’ BMI [*F*_(1,40)_ = 4.127, *p* = 0.049, *ηp*^2^ = 0.094], which importantly was further qualified by a significant interaction of physiological status, size and participants’ BMI [*F*_(1,40)_ = 6.053, *p* = 0.018, *ηp*^2^ = 0.131]. This suggested that the experimental manipulation of the physiological status had different effects on participants’ liking ratings of round and slim stimuli depending on participants’ BMIs.

**Fig. 3:**
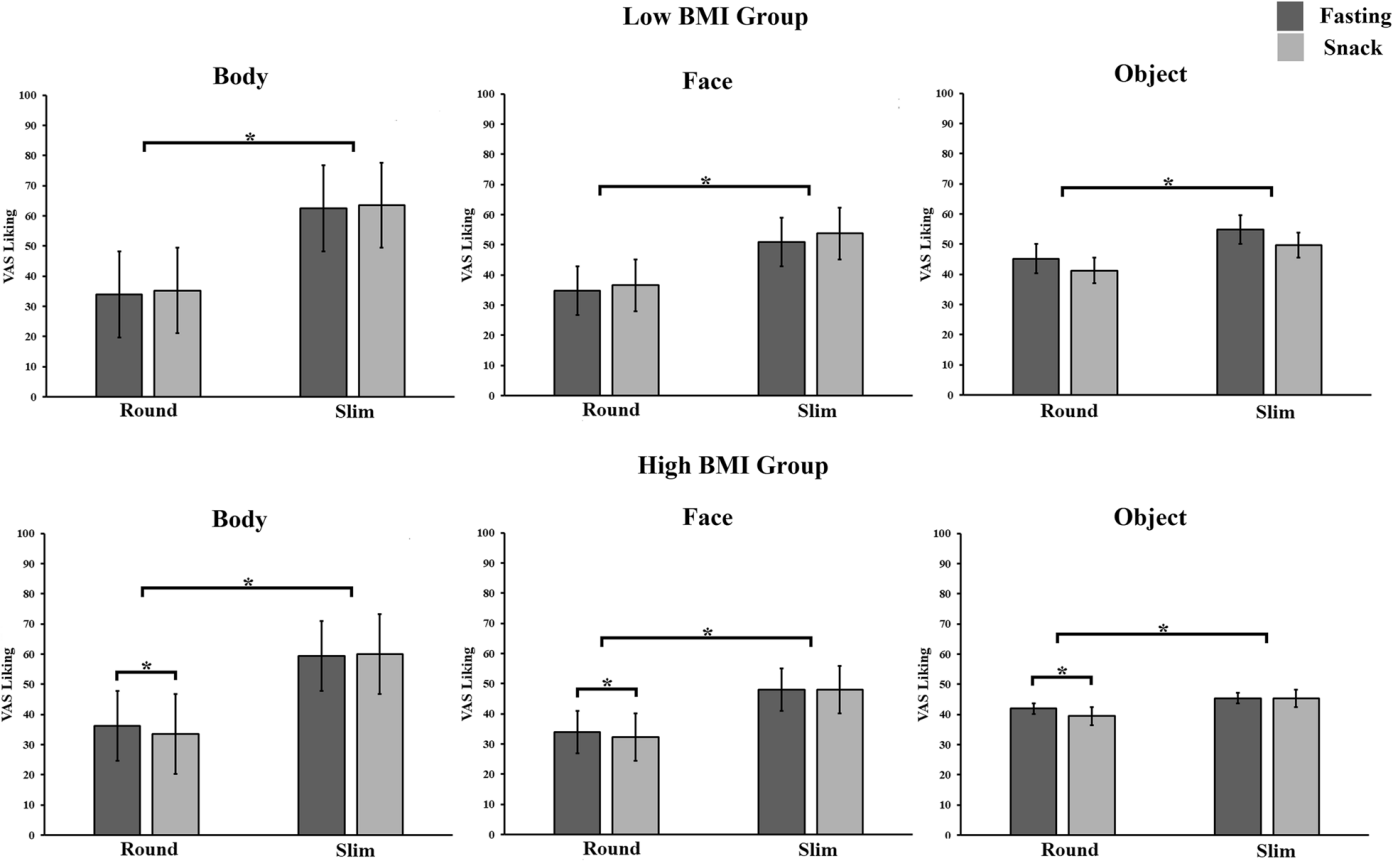
Mean (±SEM) scores of the visual analogue scale (VAS) of the aesthetic judgements for the three types of stimuli (body, face and object), the two levels of roundness (round, slim) in the two physiological conditions (fasting, snack), for participants with Low and High BMI. The asterisk symbol indicates a significant difference (*p < 0.05).

To explore the source of the significant modulation of participants’ BMI on the interaction between physiological status × size, we split the participants into those with BMI below the median (Low BMI [n = 22]; mean BMI = 20.48 kg/cm^2^ ± 0.28) and those with a BMI above the median (High BMI [n = 22]; mean = 26.02 kg/cm^2^ ± 0.43) based on the median BMI score of the whole group of participants (median BMI = 22.63 kg/cm^2^). According to the World Health Organization (WHO) BMI classification, Low BMI participants were classified as normal weight and High BMI participants were classified as overweight/pre-obese. The two groups did not differ in terms of self-reported hunger ratings after fasting (High BMI 23.30 ± 3.78 vs. Low BMI: 28.65 ± 4.46, *t*(42) = 0.92, *p* = 0.365). We then conducted two separate follow-up mixed model ANOVAs with physiological status and size as within subject variables, respectively for High vs. Low BMI participants’ groups. The ANOVAs revealed a significant interaction between the two within-subject variables only for the High BMI group [*F*_(1,20)_ = 10.647, *p* = 0.004, *ηp*^2^ = 0.347]. Importantly, post hoc analysis showed that participants with a High BMI liked more slim than round stimuli during both fasting and snack conditions (all *ps* > 0.001). On the other hand, round stimuli were liked more in the fasting (37.40 ± 1.63) than in the snack condition (35.07 ± 1.97; *p* = 0.001). No difference was observed between the liking ratings of slim stimuli in the fasting and snack conditions (50.94 ± 1.53 vs. 51.07 ± 1.92, *p* = 0.683). For the Low BMI group, a significant main effect of size was observed [*F*_(1,22)_ = 87.181, *p* < 0.001, *ηp*^2^ = 0.798]. However, neither the main effect of physiological status [*F*_(1,22)_ = 0.325, *p* = 0.574, *ηp*^2^ = 0.014] nor the interaction of physiological status × size was significant [*F*_(1,22)_ = 0.050, *p* = 0.825, *ηp*^2^ = 0.002, see Fig. 3] in the Low BMI group.

Finally, the bivariate correlation between participants’ BMI and the appetite induced change in aesthetic preference [(RoundFasting – RoundSnack) – (SlimFasting – SlimSnack)] for all three stimuli was significant (*r* = 0.34, *p* = 0.026). Accordingly, participants with higher BMI showed larger hunger-induced shift of aesthetic preference for all three types of stimuli (body, face and object).

## Discussion

In the current research, we aimed to extend and qualify previous evidence of an effect of physiological states of hunger on the aesthetic appreciation of human bodies. In particular, we tested the specificity of the increased appreciation of roundness under starvation for those stimuli that convey information about body adiposity, namely bodies and, indirectly, faces, but not for other objects, such as vases, whose size is unrelated to fat storage. To this aim, we used a within-subject design, in which the same participants rated how much they liked slim and round bodies, faces and vases after a period of 12 h fasting or after having interrupted a comparably long fasting with a (controlled) snack. Furthermore, we also controlled for individual differences in perceived hunger and BMI. Our results replicated the findings that aesthetic appreciation of human bodies can be indeed influenced by physiological states of hunger. Importantly, we added to the current picture by showing that not only bodies, but also faces, which may provide an at least indirect clue to fat storage, and objects, whose size is unrelated to fat storage, might be subjected to hunger-induced preference shifts towards roundness. Crucially, we also showed that these shifts for rounder stimuli can be influenced by individual differences in anthropometric measures of adiposity, as measured with BMI. With these regards, we found that despite all participants showing stronger liking preferences for slimmer stimuli, only individuals with High BMI exhibited a hunger-induced preference shift towards roundness. No such a shift was observed in the Low BMI group.

Several previous studies have shown that rounder bodies are rated as more attractive and liked more by hungry than satiated observers [21, 22, 26, 27]. This evidence has been interpreted within the Insurance Hypothesis [25], according to which individuals should respond to food insecurity cues, as those conveyed by hunger, by experiencing psychological and behavioural changes that promote increased fat storage. Such changes are supposed to be driven by the increased survival afforded by fat stores in buffering against energy shortfalls during times of food scarcity. Accordingly, it might be plausible that to counter possible future shortages in food supply, individuals must adapt by shifting their preference for rounder bodies under conditions of hunger. Thus, hunger-linked shifts in judgements of ideal body size are thought to favour this adaptation [21, 22, 26]. This view has been also called into action to explain cultural differences in ideal body weight [25].

Importantly, whilst the Insurance Hypothesis does not make specific claims about the specificity of the effects of food scarcity on hunger-induced shifts towards rounder faces, it can accommodate our finding that, when fasting, High BMI participants preferred rounder faces after fasting than after a snack. This does not come at surprise considering that face adiposity can influence person attractiveness as a proxy of body adiposity [32]. Indeed, several lines of research suggested that faces and bodies provide somewhat overlapping but also uniquely valuable pieces of socially relevant information. For example, studies have also shown that women’s body size, as indexed by BMI, provides significant cues of their current fertility, pregnancy status, and ability to support foetal development [43–47]. In a similar vein, regarding facial cues, women’s faces provide significant cues of their health, age, femininity, and personality traits [48–51]. Previous studies also supported the notion that both body and face equally influence judgments of women’s overall attractiveness [52, 53]. With these regards, a study by Bleske-Rechek et al. [54] reported that ratings of women’s bodily and facial attractiveness independently predicted the overall ratings of person attractiveness, thus suggesting that attributes of face and body may share some underlying factor of genetic quality that is perceived as attractive [55]. Accordingly, increased preference for rounder faces and bodies in hungrier participants might be due to the genuine (and similar) importance of bodies and faces in conveying socially relevant information which are pivotal to human physical attractiveness and human mate choice decisions.

What can more loosely fit with the Insurance Hypothesis is the finding that food depletion also had an influence on objects, and not only on bodies and faces. Indeed, participants liked more round vases under starvation than after being satiated, and the effect for objects was comparable to that for bodies and faces. This agrees with and extend to a within-subject experimental design the findings of the correlational study by Saxon and colleagues [27], which found that hungrier people were more likely to select bigger objects, and not only rounder bodies, as more attractive. Nevertheless, it is worth noticing that this study still found greater effects of perceived hunger for body stimuli, especially female bodies, than for objects. This was partially in keeping with the results of Swami et al. [26], which failed to find evidence to suggest that hunger influences judgements of other types of objects. However, this null result was not contrasted, within the same study, with a significant effect of hunger on body appreciation, thus leaving the possibility of power issues. Here, using an experimental manipulation of hunger and a similar aesthetic (liking) judgement as in the study by Swami et al. ([26], how aesthetically pleasing objects are), we found comparable hunger effects on the appreciation of both round bodies (and faces) and round objects. This keeps with a general effect of hunger to domains that are irrelevant for food intake or fat storage, as suggested by the findings that hunger gives rise to a greater desire to possess and acquire more samples of non-food objects [6]. Thus, in accordance with Saxton et al. [27], it might be that hunger motivates people towards abundance in general, explaining why our participants liked more round stimuli in general, and not only round bodies and faces, when starving than when satiated. This does not necessarily contrast with the Insurance Hypothesis, but it may qualify it. Indeed, the effects of hunger may be routed in an increased sensitivity to cues of food scarcity, but they may extend well beyond food intake and body adiposity, generally driving sensitivity to bountifulness.

In this sense, the Insurance Hypothesis might help with explaining why hunger-induced preference shifts towards roundness of all stimuli were observed in individuals with higher BMI only. Indeed, it predicts that optimal level of body fat to store depends upon security of access to food and that higher body weights should be more common when food insecurity is high because that would make body mass safe. Individuals with higher BMI might perceive greater food shortfall and, thus, stronger shift towards a preference for rounder stimuli. Nevertheless, this does not seem to be case in our study, given that lack of significant difference in self-reported hunger ratings after fasting in High vs. Low BMI groups. Yet, it might be that measurements of the subjective disposition to eat, i.e., self-reports of hunger adopted in our study, instead of other measurements of biomarkers, for e.g., blood concentrations of different hormones of satiation and satiety, might have not been sensitive enough to detect physiological perceptions of greater food shortfall in High BMI group. Accordingly, a combined approach which employs kinetics measures of satiety biomarkers and energy metabolism measures might be more suitable for quantifying this psycho-physiological construct in future [56]. Furthermore, the claim that food insecurity is a predictor of high body weights in humans is far from being conclusive. Indeed, the overall association may be moderated by differences in individuals’ socio-economic status, sex, and self-body perception [25, 31], pointing to a multifactorial mechanism.

Indeed, it has been shown that personal BMI is an important moderator of how both men and women perceive physical attractiveness of others. For example, a study by Tovee and colleagues [57] showed that the estimation of BMI and physical attractiveness is dependent on the observer’s own BMI. This way, individuals of a similar BMI are likely to perceive each other as attractive, and so there may be a positive assortment for BMI in human mate selection. Furthermore, this modulation suggests an intriguing answer to those investigations on differences in body-mass preferences for different cultures [58, 59]. For example, Polynesians have been reported to find optimally attractive a body mass heavier than comparable western populations, but also, they have been reported to have heavier personal BMI values [59]. Accordingly, effects of hunger-induced preference shift for rounder bodies in participants with higher levels of adiposity observed in our study, might be linked to a perceptual shift of rounder participants’ aesthetic preference, so that individuals with higher levels of body fatness might shift their preference towards rounder bodies in accordance with their own evaluation of personal body mass and shape.

One alternative but not mutually explanation for the moderating role of BMI could be that it reflects a decreased quantity of food that was consumed in individuals with Low vs. High BMI. This confound might indeed affect the quasi-experimental manipulation of testing participants before vs. after a self-served meal [22, 26]. However, we believe this is not the case in our study, given that the stringent design adopted by our work ensured that participants ate the maximum amount of food in a laboratory setting. Accordingly, during our experimental manipulation of snack, all participants were offered the same type and quantity of food (i.e., maximum of two bananas), which is also considered a healthy (low calories) type of food, thus allowing for a more rigorous test of factors that might impact upon people’s affective state and aesthetic appreciation.

A possible neural explanation for the current results might also refer to the contribution of the insular cortex, which represents a key region for the interpretation and integration of interoceptive signals originating from within the body [see [60] for a review], including distension of the stomach after eating, as well as appetite signalling [61]. A study by Yokum, Ng and Stice [62] has also reported a positive relationship between BMI and insula activation. Thus, the positive relationship between BMI and change in appetite-dependent aesthetic ratings towards rounder stimuli observed in our study might reflect a different degree of default insula activation, which is known to be important for self-body awareness [60].

Accurate interoceptive awareness is also relevant to cognitive and emotional facets of body image and it is accompanied by greater bodily satisfaction and identification, as well as by increased attention to physical appearance [63]. On the other end, deficits in interoception in adults’ population have been linked to higher BMI and may also contribute to increase of body weight (see [64], for an interesting systematic review of the relationship between interoception and BMI). Accordingly, interoceptive deficits could be linked to internal appetite signals that encourage satiety being less strongly weighted into eating-related decision-making [65]. If this was the case, then individuals with lower self-reported interoceptive accuracy are less likely to report eating in response to internal and satiety signals [64, 66]. According to this view, it might be that High BMI participants of our study, who presumably report lower interoceptive accuracy are then less likely to report eating in response to internal and satiety signals which in turn would affect their aesthetic judgments of rounder bodies, at a greater extent than it is the case for Low BMI group. On the other hand, higher BMI may also affect interoception because of the association of this condition with a range of changes to underlying physiology [67]. For example, obesity may result in attenuated or ‘blunted’ appetite response [68] and increased heart rate variability [69], all of which might influence interoception with a range of interoceptive signals being ‘weaker’ or more difficult to perceive among individuals with obesity. Regardless of the temporal relationships between interoception and levels of adiposity, the hypothesis of a contribution of interoceptive awareness to aesthetic judgements in High BMI individuals is intriguing. Nevertheless, our hypothesis remains speculative and future studies should assess the potential moderating role of interoceptive deficits in hunger-induced aesthetic preference shifts for body fatness in relation to individuals’ BMI.

There are some limitations to this work which warrant consideration. First, addressing the effects of gender (either of the observer or of the model) on hunger-induced shifts of aesthetic preference went beyond the scope of this investigation (and the number of participants per each gender group did not allow to test for this hypothesis). Thus, we cannot rule out that our results might be moderated by specific cognitive and neural organization of aesthetic body appreciation in male and female observers [37] or by the specific gender-typing features of the model body under evaluation [41]. Accordingly, previous works, which focussed predominantly on men’s judgements of women’s bodies, reported that the effect of hunger on judgements of women’s body fatness was more dramatic than its effect on judgements of other stimuli [27]. Although in the current study, we took advantage of the use of computer-generated images to create alterable 3-D human figure models with the standard “emaciated” and “heavy” settings supplied by the software Poser Pro 2010 (e-frontier, Santa Cruz, CA, USA), which simulate fat distribution in a realistic way as adiposity increases (see [35] for details on the creation of stimuli), at present we do not know which specific bodily and face cues of our virtual models were particularly salient to participants when they were making their aesthetic ratings. To address these issues, future work might seek to uncover whether there are specific (perhaps sexual) cues of the body or face of conspecifics that go beyond size and body shape (e.g., muscularity) that are particularly relevant to aesthetic judgements depending on one’s physiological hunger and the gender of the observers. Nevertheless, the general shift toward roundness of the aesthetic preference of both human and object stimuli seems to call into play domain-general mechanisms for the effects of hunger on aesthetic appreciation. Although one could interpret these results as evidence for ‘domain-general’ effects of psychological states of hunger on preference of bodily and object stimuli, an alternative explanation is that presentation of a vase might automatically generate a change in aesthetic perception simply because of its perceptual similarity (i.e., they share low-level sensory attributes) to the shape of a human body. We believe this is unlikely given that variation in shape (other than the size) of our vases all of which featuring different degrees of curviness, regularity, and number of acute angles, therefore not necessarily resembling that one of a human body, ensured that participants perceived body stimuli as a separate (non-corporeal) class of stimuli. However, additional research would be necessary to conclusively disentangle whether perceptual low-level similarity of body versus vase stimuli might have affected aesthetic appreciation of the external world.

One other limitation of our study is that we did not directly assess participants’ socio-economic status or their financial satisfaction/security similarly to what has been done in previous work on this topic [22, 43]. Accordingly, the study by Nelson and Morrison [22] reported that either financially dissatisfied or hungry men preferred a heavier mate than did financially satisfied men or satiated men, respectively. However, the study by Swami, Tovée and Furnham [21] failed to replicate Nelson and Morrison [22]’s findings showing that financially dissatisfied men did not rate a heavier female body weight as more attractive than did financially satisfied men. In light of these contrasting findings, we cannot exclude that other sociodemographic variables, including participants’ socio-economic status, might have affected the results of our study and future investigations are needed to better elucidate the role of financial security in shaping the aesthetic appreciation of the body depending on hunger sensation.

One further limitation of our study is that we asked participants to judge bodies, faces and objects during the same day, which might have led to response biases according to which hungrier participants provided higher aesthetic judgements to bigger bodies and faces, thus continuing this pattern of responses when they viewed objects (vases). Whilst this possibility could be only ruled out by a study during which participants are presented with all class of stimuli but in separate setting, yet we believe this not to be the case in our study for the reason that the order of presentation of the tree stimulus categories (bodies, faces and vases) was counterbalanced across participants, thus minimizing potential adaptation aftereffects or bias to systematically shift responses towards roundness across categories.

## Conclusions

We provide evidence of general effects of food depletion/intake on the aesthetic judgements of faces and objects, which go far beyond the domain of body perception. We also show that participants with high BMI might be particularly susceptible to hunger-induced preference shifts of preference for body fatness, with greater appreciation of rounder stimuli in hungrier and heavier participants. On the contrary, participants with lower levels of body adiposity (Low BMI) appeared to be more resistant to changes of their aesthetic ratings for rounder stimuli, regardless of their physiological states of hunger and satiation. Future work might seek to elucidate the relationship between physiological states of hunger and shifts in appreciation of the human bodies and whether this relationship might be mediated by individual traits associated to the beholder’s body adiposity.

## Data Availability

The dataset supporting the conclusions of this article is available in the OSF repository, https://doi.org/10.17605/OSF.IO/ZR9XE, https://osf.io/4tvm9/.
